# P-1932. Co-Administration of Triazoles with Calcineurin or mTOR Inhibitors in Solid Organ Transplant Patients Hospitalized with Invasive Aspergillosis

**DOI:** 10.1093/ofid/ofaf695.2100

**Published:** 2026-01-11

**Authors:** Barbara D Alexander, Melissa D Johnson, Belinda Lovelace, Craig I Coleman

**Affiliations:** Duke University School of Medicine, Durham, North Carolina; Duke University, Durham, North Carolina; F2G, Inc., Princeton, New Jersey; University of Connecticut School of Pharmacy, Storrs, CT

## Abstract

**Background:**

Triazoles decrease the metabolism of calcineurin and mammalian target of rapamycin (mTOR) inhibitors through CYP3A4 inhibition, resulting in increased exposure and potential for serious adverse events. The extent of co-administration of triazoles and calcineurin or mTOR inhibitors in solid organ transplantation (SOT) patients hospitalized for invasive aspergillosis (IA) in real world settings is unknown.

**Figure:**
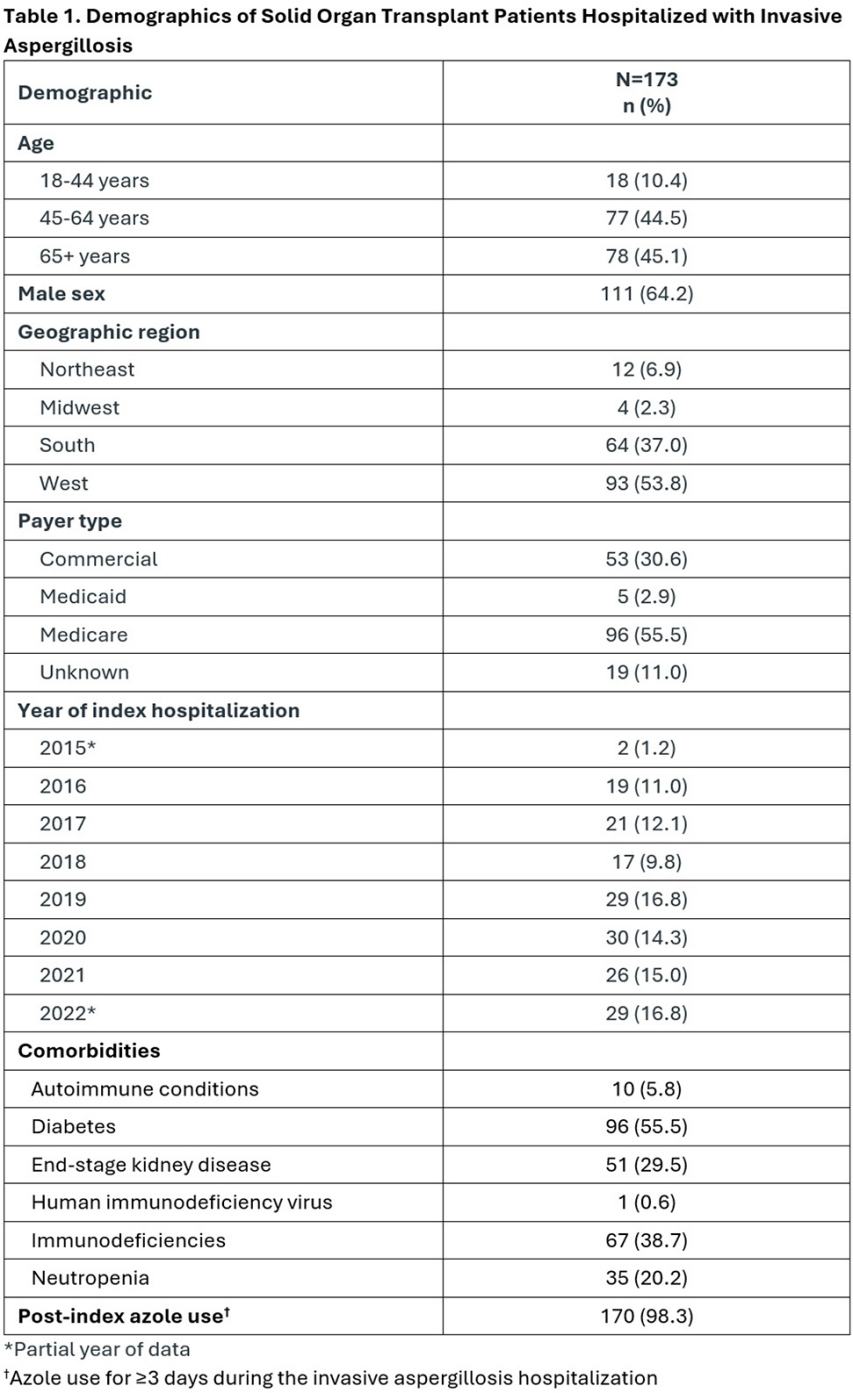

**Figure:**
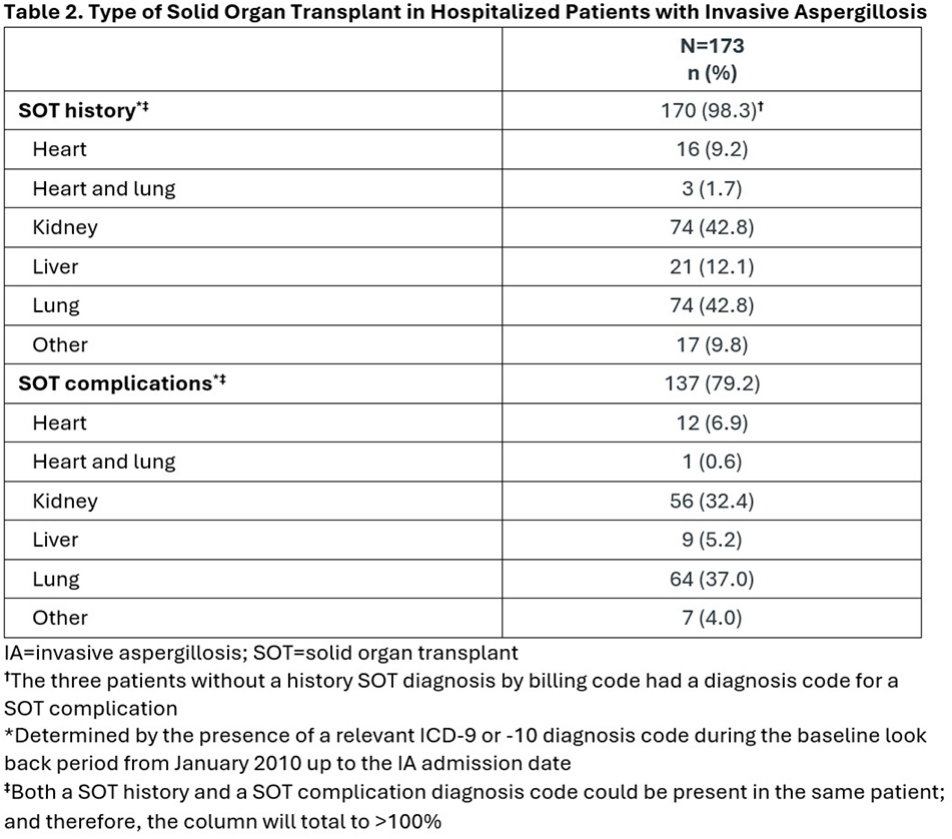

**Methods:**

This retrospective cohort study utilized United States IQVIA claims data. To be included in this study, patients had to be adults with ≥1 claim for an inpatient stay with a diagnosis code for IA (B44.0, B44.1, B44.2, B44.7) in any coding position during the patient selection period (October 2015 to November 2022) and have evidence of systemic antifungal therapy for ≥3 days during the hospitalization. This cohort was limited to patients with a history of SOT and/or SOT-related complications identified by ICD-9 or -10 codes from January 2010 up to the IA admission date (index date). The proportion of patients concomitantly receiving a triazole known to potentially cause a moderate-to-strong drug interaction with a calcineurin or mTOR inhibitor was determined.

**Figure:**
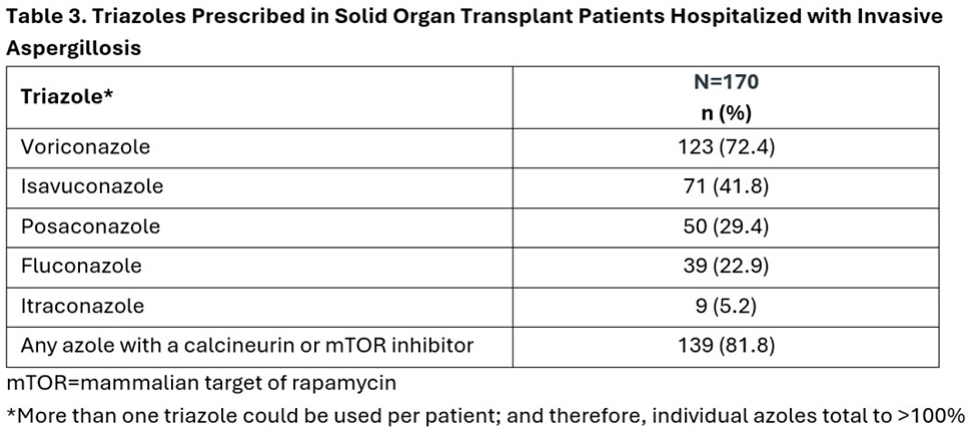

**Figure:**
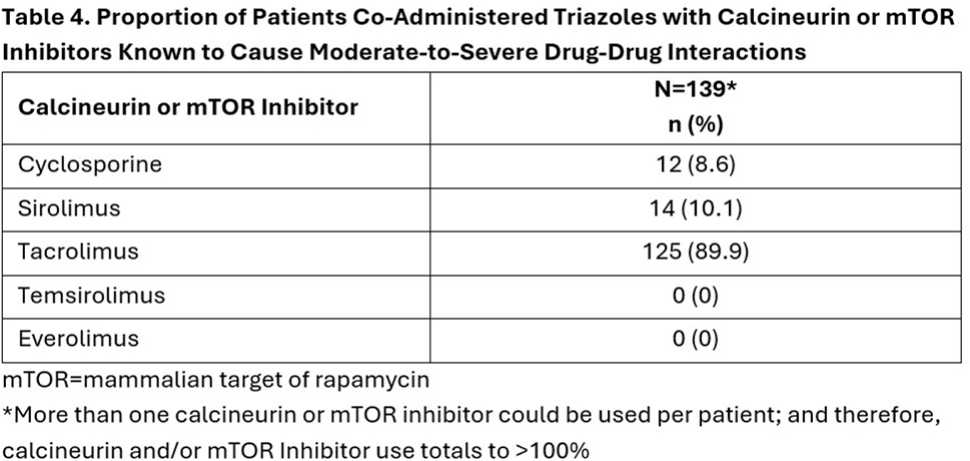

**Results:**

We identified 173 newly diagnosed IA patients with SOT, predominantly kidney and lung transplant (Tables 1 and 2). Triazoles (with or without other systemic antifungals) were used in 170 (98.3%) of these patients (Table 3) for a mean ± standard deviation duration of 116 ± 184 (median = 40) days. Voriconazole (73.2%) and isavuconazole (42.3%) were most prescribed. Calcineurin and/or mTOR inhibitors (a majority tacrolimus, 89%) were co-administered with an interacting triazole in 139 of the 170 triazole-treated cases (81.8%) (Table 4).

**Conclusion:**

The co-administration of calcineurin or mTOR inhibitors, particularly tacrolimus, with triazoles is commonplace ( >80%) in SOT patients hospitalized with IA. The calcineurin/mTOR inhibitor-triazole drug interaction has been reported to be a concern and require dose adjustment and careful therapeutic drug monitoring to reduce serious adverse events. New antifungals to treat IA without the potential for serious drug interactions with calcineurin or mTOR inhibitors are needed.

**Disclosures:**

Melissa D. Johnson, PharmD MHS AAHIVP, Biomeme: Licensed technology, method to detect fungal infection|Biomeme: Licensed technology, method to detect fungal infection|Scynexis: Grant/Research Support|Scynexis: Grant/Research Support|UpToDate: Author Royalties|UpToDate: Author Royalties Belinda Lovelace, PharmD, MS, MJ, F2G Inc.: Employee Craig I. Coleman, PharmD, F2G Inc.: Advisor/Consultant|F2G Inc.: Grant/Research Support

